# Supplementary material for: Development and validation of reliable astaxanthin quantification from natural sources
Source: PLoS One. 2022 Dec 2;17(12):e0278504. doi: 10.1371/journal.pone.0278504 (PMC9718415; doi:10.1371/journal.pone.0278504)
Supplement: S1 Table — (PDF) [file pone.0278504.s001.pdf]

S1 Table. Overview of the astaxanthin content in the various experiments.

	num-ber of trials	all-*E*-astaxanthin proportion				9*Z*-astaxanthin proportion				13*Z*-astaxanthin proportion				di-*Z*-astaxanthin proportion				Total astaxanthin	
		of biomass		of total Ax[a]		of biomass		of total Ax		of biomass		of total Ax		of biomass		of total Ax		% of biomass	
		% w/w	SD[b]	%	SD	% w/w	SD	%	SD	% w/w	SD	%	SD	% w/w	SD	%	SD	% w/w	SD
**Detection limits and linearity of astaxanthin standards**																			
Ax standard in acetone	29	-	-	98.0	0.2	-	-	2.4	0.2	-	-	0.4	0.09	-	-	0.2	0.03	-	-
Ax standard in extraction phase	9	-	-	92.8	0.3	-	-	5.9	0.5	-	-	1.3	0.5	-	-	-	-	-	-
Ax-Mp[c] in extraction phase	8	-	-	93.0	0.7	-	-	4.9	0.3	-	-	2.1	0.9	-	-	-	-	-	-
**Enzymolysis with cholesterol esterase**																			
2.0 U	3	3.6	0.05	86.4	0.7	0.2	0.01	4.9	0.2	0.19	0.02	4.5	0.5	0.2	0.01	4.2	0.3	4.2	0.03
1.5 U	3	3.5	0.06	86.3	0.6	0.2	0.00	5.0	0.1	0.18	0.02	4.4	0.5	0.2	0.01	4.4	0.2	4.0	0.06
1.0 U	2	3.5	0.01	86.3	0.3	0.2	0.04	5.0	0.3	0.18	0.01	4.4	0.4	0.2	0.01	4.3	0.3	3.6	0.01
0.5 U	3	3.2	0.1	87.3	0.1	0.2	0.01	4.8	0.2	0.14	0.01	3.9	0.1	0.2	0.01	4.1	0.09	3.7	0.1
0.1 U	3	1.5	0.5	87.4	0.3	0.08	0.03	4.5	0.2	0.06	0.02	3.6	0.2	0.08	0.02	4.6	0.3	1.8	0.6
0.05 U	3	0.8	0.3	87.8	0.3	0.04	0.01	4.4	0.1	0.03	0.01	3.6	0.2	0.04	0.01	4.3	0.2	0.9	0.3
0.5 U and 1.5 h incubation	3	3.4	0.1	88.3	0.2	0.2	0.01	4.3	0.2	0.15	0.01	3.8	0.3	0.1	0.01	3.6	0.1	3.8	0.1
2.0 U and 1.5 h incubation	3	3.6	0.1	88.3	0.9	0.2	0.01	4.1	0.07	0.17	0.02	4.2	0.5	0.1	0.02	3.5	0.3	4.1	0.2
**Processing of liquid-liquid extracts**																			
AX standard in extraction phase	4	-	-	94.9	0.8	-	-	4.5	0.8	-	-	0.7	0.1	-	-	-	-	-	-
AX standard, aliquot in acetone	4	-	-	97.1	0.4	-	-	1.5	0.02	-	-	1.1	0.3	-	-	0.3	0.07	-	-
AX standard, all in acetone	3	-	-	96.9	0.3	-	-	1.5	0.01	-	-	1.3	0.2	-	-	0.3	0.05	-	-
**Detection limits and linearity of astaxanthin determination**																			
0.04 mg *H. pluvialis*	3	3.3	0.05	88.2	0.8	0.1	0.002	2.9	0.07	0.2	0.01	5.1	0.2	0.2	0.03	3.8	0.6	3.8	0.06
0.2 mg *H. pluvialis*	3	3.7	0.06	89.0	0.6	0.1	0.004	3.0	0.1	0.2	0.02	4.6	0.4	0.1	0.03	3.4	0.7	4.1	0.04
0.4 mg *H. pluvialis*	3	3.7	0.01	88.9	0.7	0.1	0.01	2.9	0.3	0.2	0.02	4.4	0.5	0.2	0.01	3.8	0.3	4.1	0.03
0.8 mg *H. pluvialis*	3	3.7	0.01	87.9	0.4	0.1	0.002	3.0	0.06	0.2	0.01	5.2	0.3	0.2	0.01	4.9	0.2	4.3	0.03
1.2 mg *H. pluvialis*	3	3.7	0.07	86.8	1.9	0.2	0.008	3.5	0.2	0.2	0.05	4.9	1.1	0.2	0.03	4.8	0.6	4.3	0.06
1.6 mg *H. pluvialis*	3	3.6	0.03	85.4	1.3	0.1	0.005	3.3	0.05	0.3	0.03	6.3	0.5	0.2	0.04	5.1	0.8	4.3	0.08
2.0 mg *H. pluvialis*	3	3.6	0.02	85.7	1.2	0.1	0.002	3.4	0.07	0.3	0.03	6.1	0.6	0.2	0.03	4.8	0.7	4.2	0.03
3.3 mg *H. pluvialis*	1	3.2	-	75.7	-	0.3	-	7.1	-	0.3	-	7.7	-	0.4	-	9.4	-	4.3	-
4.0 mg *H. pluvialis*	1	2.9	-	74.9	-	0.3	-	6.6	-	0.3	-	8.1	-	0.4	-	10.4	-	3.8	-

**Precision of astaxanthin determination**																			
A	5	3.5	0.04	80.4	0.8	0.2	0.01	5.0	0.3	0.3	0.03	6.9	0.6	0.3	0.01	7.7	0.3	4.3	0.04
B	4	4.0	0.04	81.3	0.6	0.3	0.02	5.0	0.3	0.4	0.02	7.8	0.4	0.3	0.01	5.9	0.1	4.9	0.06
***H. pluvialis* liquid cultures**																			
M1	3	3.8	0.2	91.0	0.7	0.1	0.004	3.0	0.05	0.1	0.01	3.4	0.3	0.1	0.01	2.7	0.3	4.2	0.1
M2	5	2.4	0.1	84.1	2.1	0.1	0.02	4.3	0.7	0.1	0.02	3.9	0.4	0.2	0.03	7.7	0.9	2.8	0.1
M3	3	2.3	0.04	83.6	0.08	0.1	0.03	5.0	0.2	0.1	0.02	3.8	0.09	0.2	0.03	7.6	0.2	2.8	0.5
M4	3	0.7	0.04	77.7	1.9	0.03	0.002	3.6	0.3	0.04	0.004	4.2	0,5	0.1	0.008	14.5	1.2	0.9	0.02
**Oleoresins**																			
O1	3	5.4	0.4	78.8	1.2	0.4	0.02	5.6	0.04	0.3	0.04	4.9	0.4	0.7	0.05	10.8	1.0	6.9	0.4
O2	7	1.3	0.03	82.7	1.2	0.08	0.003	5.1	0.2	0.06	0.001	3.7	0.2	0.1	0.02	8.5	1.4	1.6	0.04
O3	3	3.4	0.3	60.1	1.8	0.7	0.1	13.0	0.4	0.4	0.08	7.2	0.6	1.1	0.2	19.7	0.8	5.7	0.7
**Method comparison to photometric astaxanthin extraction**																			
Batch A, day 22	1	1.0	-	88.2	-	0.04	-	3.5	-	0.04	-	3.5	-	0.05	-	4.8	-	1.1	-
Batch A, day 23	1	1.3	-	88.3	-	0.05	-	3.7	-	0.04	-	3.7	-	0.06	-	4.3	-	1.4	-
Batch A, day 24	1	1.9	-	87.7	-	0.09	-	4.2	-	0.08	-	3.8	-	0.09	-	4.3	-	2.1	-
Batch A, day 27	1	2.3	-	88.3	-	0.1	-	4.2	-	0.1	-	3.9	-	0.09	-	3.6	-	2.6	-
Batch A, day 28	1	2.5	-	88.8	-	0.1	-	3.9	-	0.1	-	3.9	-	0.09	-	3.6	-	2.8	-
Batch B, day 22	1	1.3	-	87.6	-	0.05	-	3.5	-	0.05	-	3.5	-	0.08	-	5.4	-	1.5	-
Batch B, day 23	1	1.8	-	87.8	-	0.08	-	3.8	-	0.07	-	3.7	-	0.09	-	4.7	-	2.0	-
Batch B, day 24	1	1.9	-	87.7	-	0.09	-	4.1	-	0.08	-	3.8	-	0.09	-	4.4	-	2.1	-
Batch B, day 27	1	2.5	-	88.3	-	0.1	-	3.9	-	0.1	-	3.9	-	0.1	-	4.0	-	2.9	-
Batch B, day 28	1	2.7	-	88.0	-	0.1	-	4.0	-	0.1	-	3.9	-	0.1	-	4.2	-	3.1	-
**Shelf life of lyophilized and undisrupted H. pluvialis biomass partially exposed to ambient atmosphere**																			
0 days at ambient atmosphere	2	0.8	0.2	80.8	0.003	0.04	0.004	3.8	0.4	0.03	0	3.2	0.08	0.1	0	12.3	0.5	1.0	0.01
7 days at ambient atmosphere	1	0.7	-	80.3	-	0.03	-	4.1	-	0.02	-	2.8	-	0.1	-	12.8	-	0.8	-
104 days at ambient atmosphere	1	0.4	-	78.9	-	0.02	-	3.9	-	0.01	-	2.5	-	0.08	-	14.8	-	0.5	-
489 days at ambient atmosphere	1	0.3	-	80.8	-	0.02	-	3.9	-	0.01	-	2.6	-	0.05	-	12.7	-	0.4	-

**Dried and disrupted *H. pluvialis* biomass vacuumed and exposed to ambient atmosphere**																			
Opened	5	3.5	0.04	80.4	0.8	0.2	0.01	5.0	0.3	0.3	0.03	6.9	0.6	0.3	0.01	7.7	0.3	4.3	0.04
Closed	4	4.0	0.04	81.3	0.6	0.3	0.02	5.0	0.3	0.4	0.02	7.8	0.4	0.3	0.01	5.9	0.1	4.9	0.06

[a]Ax = Astaxanthin

[b]SD = Standard deviation

[c]Ax-Mp = Astaxanthin monopalmitate
