# Supplementary material for: Development and validation of reliable astaxanthin quantification from natural sources
Source: PLoS One. 2022 Dec 2;17(12):e0278504. doi: 10.1371/journal.pone.0278504 (PMC9718415; doi:10.1371/journal.pone.0278504)
Supplement: S2 Table — (PDF) [file pone.0278504.s002.pdf]

S2 Table. Overview of the astaxanthin content determined in ethanolic SC-CO_2 extracts.

	number of trials	all-*E*-astaxanthin proportion				9*Z*-astaxanthin proportion				13*Z*-astaxanthin proportion				di-*Z*-astaxanthin proportion				Total astaxanthin	
		in extract		of total Ax[a]		in extract		of total Ax		in extract		of total Ax		in extract		of total Ax		in extract	
		µg/mL	SD[b]	%	SD	µg/mL	SD	%	SD	µg/mL	SD	%	SD	µg/mL	SD	%	SD	µg/mL	SD
**Ethanolic oleoresins**																			
1-ethanol present	5	52.9	0.38	86.7	1.0	2.4	0.1	3.9	0.3	2.1	0.1	3.4	0.1	3.7	0.8	6.0	1.1	61.0	1.0
1-ethanol evaporated	2	53.1	0,46	84.1	0.2	2.9	0.1	4.6	0.2	2.3	0.1	3.6	0.1	4.9	0.2	7.7	0.3	63.2	0.7
2-ethanol present	3	8.6	0.22	89.1	0.9	0.3	0.05	3.2	0.4	0.3	0.02	3.4	0.1	0.4	0.1	4.3	0.4	9.7	0.3
2-ethanol evaporated	2	8.8	0.21	85.0	0.3	0.5	0.03	4.5	0.4	0.4	0.01	3.5	0.2	0.7	0.04	7.0	0.2	10.3	0.2

[a]Ax = Astaxanthin

[b]SD = Standard deviation
