# Supplementary material for: Development and validation of reliable astaxanthin quantification from natural sources
Source: PLoS One. 2022 Dec 2;17(12):e0278504. doi: 10.1371/journal.pone.0278504 (PMC9718415; doi:10.1371/journal.pone.0278504)
Supplement: Attachment — Submitted filename: Comments.docx [file pone.0278504.s003.docx]

Comments:

Sentence 41-43: “calibration……5%” seems incomplete and complicated statement.

Sentence 44: Rewrite it, as it seems the two di-Z forms are 9Z and 13Z mentioned in the sentence and if so why to mention the “two di-Z forms” in the same sentence again?

Sentence 47-49: only the dried biomass was processed for Astaxanthin and not the liquid culture or the fresh biomass?

Sentence 49-50: Cholesterol esterase (1.0 to 2.0 U) is per how many mg or mL of dried biomass or culture respectively?

Sentence 50-52: This line is not necessary here; it should be discussed in introduction section.

Sentence 53: “The reliability of photometric Astaxanthin estimation was assessed”, how? Name the method or explain in one sentence.

It’s not clear in the abstract if the authors are comparing two methods of extraction or estimation or both, please clarify.

Sentence 61-63: needs reference.

Sentence 68-69: what about the allergenicity of Astaxanthin if provided as food supplement.

Sentence 84-89: it gives explanation of why this technique is needed. The limitations due to which related techniques like this have not been used should also be stated.

Sentence 100: which wavelength?

Sentence 119-128: it gives the glimpse of steps involved, but in the introduction it was claimed that it is a shorter and faster method. However, Astaxanthin extraction, enzymolysis, liquid-liquid extraction and then analysis with UHPLC,UV/VIS spectrophotometry does not seem to be a short method.

Sentence 156: what was the make of beat miller, diameter of zirconium beads, time of beat milling, and the cooling time in the process?

Sentence 167: is the fresh biomass dewatered?

Sentence 172: Mention 3.3U/mL as cholesterol esterase concentration in abstract used for de-esterification.

Sentence 211: how the proportion of 69.4% was assumed?

Sentence 215: replace solved to dissolve.

Sentence 223: is 45 min. (0.75 h) is the minimum time used, why authors did not try the lower incubation period or higher incubation period than to 1.5 h.

Sentence 260-265: previously, it was mentioned that additional incubation time of 1.5 h was also used so; for detection limit and linearity, why only one incubation time is used?

Sentence 267-271: there is lot of confusion between the samples (fresh or dry), if fresh then how it was weighed and what is the incubation period in no. of days decided on what basis, which two samples were chosen and why?

I cannot follow the series of experiments performed and what samples were used what was the incubation period, was there any stress? I suggest authors to prepare an experimental design section with text and detail figure to explain precisely how the experiments are performed with each step and from which step sample were taken for which analysis and why?

Sentence 287: On what basis it was considered 4.5 % w/w of the *H. pluvialis* is Astaxanthin?

Sentence 300: In sentence 270-271, four days are mentioned in sentence 300 five days are mentioned, why? It’s confusing to follow.

In figure 5: It seems like there is no effect of increasing the biomass (mg) but, how it is possible if considered 4.5 % w/w of the *H. pluvialis* is Astaxanthin?

In figure 7: give legends with in the graph for samples like M1, M2 ETC.

In figure 9: which stress is imposed and why? Also indicate level of significance in each figure.

Conclusion is too long; make it precise and conclusive in 10-12 sentences. For instance sentence 986 seems like repetition of first line of conclusion. Comparison should be a part of discussion. Similarly sentence 990-991 should be discussed in methodology section.
