# Supplementary material for: Development and validation of reliable astaxanthin quantification from natural sources
Source: PLoS One. 2022 Dec 2;17(12):e0278504. doi: 10.1371/journal.pone.0278504 (PMC9718415; doi:10.1371/journal.pone.0278504)
Supplement: Attachment — Submitted filename: Reviewer comments PONE-D-22-07202.docx [file pone.0278504.s004.docx]

**Comments:**

1. In line no 44-45 sentence “In extracts from H. pluvialis, the observed measurement range was extended to 30 µg/mL”. What was the amount of *H. pluvialis* biomass taken? It should be mentioned in the abstract also.
2. Line no 47, 48, 48 ‘The precision of all-E-astaxanthin quantification in dried *H. pluvialis* biomass was calculated with a coefficient of variation of maximal 1.1%, whereas it was below 10% regarding the diastereomers” What was the amount of *H. pluvialis* biomass?
3. In the beginning sentence of introduction “Astaxanthin (3,3´-dihydroxy-β,β´-carotene-4,4´-dione) is a secondary ketocarotenoid. It has a hydrocarbon backbone that comprises a central, delocalized π-electron system. β-ionone rings terminate the hydrocarbon chain at both ends. The presence of one hydroxy- and one oxo-group at each of these terminal rings further classify it as xanthophyll.” Add reference and for more information prefer the suggested article “Light modulates transcriptomic dynamics upregulating astaxanthin accumulation in *Haematococcus*: A review” <https://doi.org/10.1016/j.biortech.2021.125707>
4. Add new references from line no 84-89.
5. Line no 157, what was the percentage of acetone used for astaxanthin extraction? Likewise line no 174 mention the percentage of petroleum ether?
6. Line no 161 please check whether it is 10.000 x g or 10,000 x g. See at other places also.
7. Mention makes and model of the instruments/ equipments used during the experiment. For instance, vortex and ultrasonicate.
8. Line no 190 and 191 ‘Optical spectra were measured in a range of 200 to 800 nm, and astaxanthin data were analyzed and quantified at 474 nm. Lutein was quantified at 448 nm.’ Add the references for astaxanthin and lutein as well, to increase the novelty of the article.
9. Line no 215 please check whether “solved” is the right word to use here. Check in the entire manuscript.
10. Check for typographical errors in the entire manuscript.
11. Measuring units should be written clearly for instance see line no 221-224.
12. Text in the conclusion should be reduced to 8-12 lines.
